# Weekly minutes of moderate to vigorous physical activity is associated with movement quality in overweight and obese older adults

**DOI:** 10.21203/rs.3.rs-3200471/v1

**Published:** 2023-07-25

**Authors:** Julie Rekant, April Chambers, Anisha Suri, Andrea Hergenroeder, Ervin Sejdic, Jen Brachs

**Affiliations:** University of Pittsburgh

**Keywords:** physical activity, body composition, aging, biomechanics, functional mobility

## Abstract

**Background:**

Physical activity can improve function and decrease healthcare spending among overweight and obese older adults. Although unstructured physical activity has been related to cardiometabolic improvements, the relationship between unstructured activity and movement quality is unclear.

**Aims:**

This study aimed to evaluate the association of amount of unstructured free-living moderate-vigorous physical activity (MVPA) with measures of movement quality in overweight and obese older adults.

**Methods:**

The association of MVPA with movement quality was assessed in 165 overweight and obese older adults (Age: 77.0(8.0) years; Body mass index (BMI): 29.2(5.3) kg/m^2^). Participants performed overground walking, the Figure of 8 Walk test, and the Five-Times Sit to Stand. Weekly physical activity was measured using a waist-worn Actigraph activity monitor.

**Results:**

Movement quality during straight path (gait speed (ρ = 0.30, *p* < 0.01), stride length (ρ = 0.33, *p* < 0.01), double-limb support time (ρ=−0.26, *p* < 0.01), and gait symmetry (ρ = 0.17, *p* = 0.02)) and curved path (F8W time (ρ=−0.22, *p* < 0.01) and steps (ρ=−0.22, *p* < 0.01)) walking were associated with weekly minutes of MVPA after controlling for age. Five-Times Sit to Stand performance was not significantly associated with weekly minutes of MVPA (ρ=−0.10, *p* = 0.13).

**Conclusions:**

Older adults with high BMIs who are less active also demonstrate poorer movement quality which should be targeted in interventions to promote healthy aging, decrease falls, and delay disability development. Future work should explore if these associations are observed in middle-aged adults so targeted interventions can be implemented even earlier in the disability development continuum.

## 1. Introduction

Over ninety percent of adults over the age of 60 in the United Stated are overweight or have obesity.[1] This means there are approximately 48 million Americans dealing with the deleterious effects of both aging and an elevated body mass index (BMI).[2] Overweight and obese older adults experience a poorer quality of life than their normal weight counterparts and have a higher incidence of disability and injury compared to those with lower BMIs.[3] As a result, caring for older adults with higher BMIs costs an additional $2,00–3,000 annually when compared to care of similarly aged normal weight adults.[4–6]

The independent effects of aging and BMI on movement quality have been well described. Older adults exhibit less symmetric gait[7] compared to young individuals,[8–10] and adults with high BMIs take shorter steps and spend more time in double support than normal weight adults.[11] Together, BMI and aging effects lead to slower,[12, 13] less efficient[14] gait and implementation of compensatory strategies for functional mobility[15] among older adults with higher BMIs. Asymmetric gait, shortened step length, and more time in double support during gait are associated with greater fall-risk,[16–20] poorer overall dynamic balance,[8, 21] and decreased walking skill.[22] Risk of sarcopenia and osteopenia increase with aging[23] and can be exacerbated by weight loss.[24] Therefore, promoting increased and better-quality movement should be emphasized in older adults.[23] Cooper et al. posited older adults with high BMIs “may require targeted interventions and additional support” for mobility and physical activity improvements.[25]

Increasing physical activity participation has been proposed as a means to decrease health care expenditures[6, 26] and promote healthy aging[27–29]. Increased physical activity participation can also improve cardiorespiratory fitness and metabolic health, protect against morbidity and mortality,[30, 31] and promote higher health-related quality of life[32, 33] in aging adults and adults with high BMIs. *Structured* exercise interventions in older adults and adults with high BMIs have been shown to be effective at improving gait and balance.[34, 35] However, the relation of movement quality specifically from gait, transfers, and curved paths to *unstructured* physical activity[36] among overweight and obese older adults has not been well described. Promoting increased, unstructured physical activity throughout the day has been proposed as a more feasible and effective way to increase overall activity in aging adults when compared to long term adherence to structured exercise programs.[37] The present analysis explores the association of physical activity and movement quality in a subpopulation of older adults particularly vulnerable to functional decline, disability development, and increased healthcare expenditure; those with high BMIs.

The goal of this analysis is to evaluate the association of the amount of unstructured free-living moderate-vigorous physical activity with measures of movement quality in overweight and obese older adults. It is expected that overweight and obese older adults who participate in greater amounts of physical activity will exhibit better walking and balance abilities compared to less active individuals, indicating the importance of physical activity participation for movement quality in a vulnerable population, and not just weight loss. The results of this analysis will inform interventions aimed at increasing activity, decreasing falls, and delaying disability development in older adults with overweight and obesity.

## 2. Methods

### 2. 1. Study Design and Participants

Participants were recruited through the University of Pittsburgh Pepper Center Registry, a list of older adults who have consented to be contacted for mobility research studies.[38] Community-dwelling older adults were included in this study if they were 65 years of age or older and ambulatory without an assistive device nor assistance of another person; the present analysis included only participants with BMIs of 26 kg/m^2^ or greater indicating they were overweight or had obesity. Participants were excluded if they experienced pain that limited their ability to participate in the study procedures or had a history of orthopedic, neurological, or visual impairment diagnoses which made participation in the study procedures unsafe. This cross-sectional study examined older adults who participated in a larger intervention trial (NCT02663778).[38] Only baseline measures are included in the present analysis. All study activities were approved by the University of Pittsburgh Institutional Review Board and participants provided written informed consent prior to completion of study activities. A trained research physical therapist conducted study visits at the University of Pittsburgh Physical Therapy – Clinical and Translational Research Center (PT-CTRC). Age, sex, and self-reported height and weight were collected.

### 2.2. Outcomes

#### 2.2.1. Self-reported physical functioning

All participants completed the Late-Life Function & Disability Instrument (LLFDI), a self-report measure with functional and disability subscales[39] shown to be valid and reliable for assessing difficulty performing physical tasks and participating in life activities in community-dwelling older adults.[40] The overall function (LLFDI – Function) and disability limitation (LLFDI – Disability) dimension subscales were included in this analysis, scaled to score out of 100 with higher scores indicating better function.[40]

#### 2.2.2. Measures of movement quality

Gait measures: Participants walked at their self-selected speeds over an instrumented walkway (Protoknetics LLBC, Haverton, PA, USA) and then completed overground walking while wearing a triaxial accelerometer (Actigraph LLC, Pensacola, FL, USA) on the low back near L3.[38] Gait speed, stride length, and double limb support time were calculated from the instrumented walkway, and gait symmetry using harmonic ratios (HRs) were computed from the accelerometry data in the medial-lateral (HR-ML), vertical (HR-V), and anteroposterior (HR-AP) directions.[7] Harmonic ratio (HR), a measure of step-to-step symmetry,[7] is used to define smoothness of walking and overall dynamic balance.[8, 21] Older adults exhibit lower HRs compared to young individuals[8–10] which has been associated with greater fall-risk. [16–20] Five-time sit to stand: Participants also completed the five-time sit to stand (5xSTS) test.[41, 42] The 5xSTS test is a functional assessment of the ability to perform a sitting to standing transfer[43] which is shown to be valid and reliable at identifying community dwelling older adults at risk for falls.[44, 45] Figure of 8 walk test: Finally, participants completed the Figure of 8 Walk test (F8W).[46] The F8W is an assessment of curved and straight path walking to characterize complex walking abilities encountered in daily life.[46] It can be used to assess gait disorders in patients with otherwise good straight-path walking abilities.[47–49] Taking more time and/or steps to complete the F8W[50] has been associated with greater fall history[51] and can be used to identify individuals with poorer mobility and balance.[35]

#### 2.2.3. Physical activity

Participants were instructed to wear an Actigraph GT3X100 (Actigraph LLC, Pensacola, FL, USA) waist-worn accelerometer during waking hours for seven consecutive days.[38] Participants with at least four days of Actigraph observation were included in the analysis per the National Health and Nutrition Examination Survey (NHANES) processing guidelines.[52] Weekly moderate-vigorous physical activity (MVPA) minutes were computed for all participants with no minimum bout length[53] using Actigraph processing thresholds established for older adults.[54, 55]

### 2.3. Data Analysis

Data were checked for normality and equal variances; data did not satisfy these conditions. Medians and interquartile ranges (IQR) were calculated to describe participant demographics. One-tailed non-parametric Spearman’s rank correlations controlling for age were computed between measures of movement quality and weekly minutes of MVPA. Gait speed, stride length, and HRs were expected to be positively associated with MVPA minutes while double limb support time, F8W time, F8W steps, and 5xSTS time were expected to be negatively associated with MVPA minutes. Age was controlled for in these correlations because of the known effect of aging on movement quality measures.[8–10, 44, 45, 56–58] Alpha was set to 0.05 for statistical significance. Analyses were performed in SPSS Version 28 (IBM, Armonk, NY, USA). Post-hoc power calculations were performed with G*Power 3.1.9.7 (Heinrich Heine University Düsseldorf, Düsseldorf, Germany).

## 3. Results

After screening, 181 participants met the inclusion criteria for the present analysis. Of those, twenty-five individuals had incomplete or missing physical activity data so 156 participants were included in the final analysis. There were no differences in demographics and movement quality metrics between those with missing physical activity data as those included in the final analysis. Participants were, on average, 77 years old, had a BMI of 29 kg/m^2^, and reported some functional difficulties (LLFDI – Function median score: 59.9) but minimal limitation due to disability (LLFDI – Disability median score: 77.6). Detailed participant demographics can be found in Table 1. The Centers for Disease Control and Prevention (CDC) and World Health Organization (WHO) recommend adults participate in at least 150 minutes of weekly MVPA.[59, 60] Adults participating in less than 90 minutes of weekly MVPA are considered inactive.[61] According to these criteria, about half of the participants in this analysis (n = 80 (51.3%)) were sufficiently active (≥ 150 minutes/week MVPA), participating in an average (IQR) of 302.2 (251.8) minutes of MVPA per week. One fifth (n = 29 (18.6%)) of the participants were somewhat active (90–149 minutes/week MVPA) and participated in, on average, 119.0 (28.4) minutes of MVPA per week. One third of participants (n = 37 (30.1%)) were inactive (< 90 minutes/week MVPA) and participated in 49.1 (34.0) minutes of MVPA per week.

Participants walked with an average gait speed of 1.08 m/s, took 9.56 seconds to complete the F8W, and took 12.96 seconds to complete the 5xSTS. Detailed information on participant performance on movement quality metrics are presented in Table 2.

Spearman’s rank correlations between weekly minutes of MVPA and movement quality measures controlling for age are shown in Table 3. Gait speed, stride length, double-limb support time, HR-V, HR-AP, time to complete the F8W, and steps to complete the F8W were all significantly associated with minutes of MVPA per week after controlling for age. These associations were small (0.1–0.3) [62]. All associations existed in the expected directions. Post-hoc power was greater than 0.87 for gait speed, stride length, double support time, F8W time, and F8W steps.

## 4. Discussion

More than 40 million Americans over the age of 60 are overweight or have obesity.[1, 2] Physical activity can be beneficial for older adults and adults with high BMIs,[30–32, 63–65] however how it is associated with movement quality in overweight and obese older adults is not well described. This analysis examined the association of movement quality with weekly minutes of MVPA in overweight and obese older adults. Weekly minutes of MVPA was significantly associated with measures of movement quality in overweight and obese older adults, even after accounting for the effects of age (Table 3).

The participants in this study reported scores on the LLFDI-Disability (77.6) consistent with having slight (73.8) to no (82.5) functional limitations due to disability.[39] However, participants scored lower on the LLFDI-Function (59.9) with scores similar to those experiencing slight (65.6) to moderate (53.2) limitations in physical functioning.[39] A score at or above 70 on the LLFDI-Disability is consistent with being able to participate in “active recreation”, while a score of 60 on the LLFDI-Function is consistent with being able to climb stairs while carrying objects and “run to catch a bus” but not walk a mile at a brisk pace, hike, nor run.[39] Nonetheless, just over half of the participants in this analysis met the CDC and WHO recommendations[59, 60] for weekly physical activity participation and another 20% were somewhat active. These rates of activity are similar to those reported for adults over 65 years old in the United States in 2021,[66] supporting that these participants are representative of the general population.

Participants in this analysis had desirable gait speeds (> 1.0 m/s) and are considered community ambulators,[67, 68] however they took shorter strides than similarly aged overweight adults[69–72] and spent more time in double support during each gait cycle[70, 73] (Table 2). Adults with obesity are known to take shorter strides and spend more time in double-limb support than their normal weight counterparts, [74, 75] however this cohort also demonstrated decreased gait quality measures compared to overweight older adults possibly related to the functional limitations reported by these study participants. As expected, gait speed, stride length, and double-limb support time were all significantly and positively associated with physical activity (Table 3), indicating the gait quality impairments which exist among adults with high BMIs are more strongly present in less active individuals. Walking slower, taking shorter steps, and spending more time in double support is thought to be a protective compensation for poor dynamic balance[76–78] and can be thought of as adopting “less risky” gait. However, gait speed has been shown to predict disability over ensuing years.[79] As such, a positive association between gait speed and minutes of MVPA after controlling for age (Table 3) adds to existing literature which supports physical activity for better health-related outcomes in older adults[80–82] independent of age effects.

The participants in the present analysis had less symmetric gait evidenced by smaller HRs in all three planes when compared to slightly older adults (82 years old vs. our cohort: 77 years old) walking at their preferred speeds.[56] Gait symmetry also showed significant associations with minutes of weekly physical activity in the vertical and antero-posterior directions after controlling for age in this analysis, however these analyses only achieved 52–65% power to assert these claims (Table 3). HR-AP and HR-V represent step to step symmetry, while HR-ML represents stride to stride symmetry.[7, 56] Several studies have demonstrated that lower HR, particularly in the antero-posterior and vertical directions, is associated with greater fall-risk in older adults.[16–20] Our findings are consistent with those of Abel et al. who found greater HR-AP to be associated with more walking activity.[83] Suri et al. also found greater HR-AP to be associated with a greater life-space.[84] This work in conjunction with our findings supports that more active older adults also have more symmetric gait, are at decreased risk for falls, and occupy a greater life space independent of age. Future work should explore this association with a larger sample.

This cohort also demonstrated impaired movement quality when curved path walking was assessed. Overweight and obese older adults in this analysis took almost 10 seconds to complete the F8W and did so with 17 steps (Table 2), indicating poor mobility and balance.[35] F8W performance has been shown to predict future functional status in older adults[85] and is associated with fall history.[51] Performance on the F8W was significantly and negatively associated with minutes of weekly physical activity after controlling for age (Table 3) demonstrating those who were more active also completed the test faster and with fewer steps regardless of age, and are at decreased risk for functional decline.

The average 5xSTS time in this cohort (12.9 sec) is both greater than the expected time to complete this assessment for similarly aged community-dwelling adults[86] and above the fall-risk cut score (12 sec). [44] Older adults with higher BMIs complete a repeated chair stand test slower than their normal weight counterparts,[87] a metric which has been used to identify older adults in need of further follow up[44] and those at risk for recurrent falls.[45] However, 5xSTS time was not significantly associated with minutes of weekly physical activity (Table 3) suggesting amount of physical activity is not associated with functional transfer abilities in overweight and obese older adults after controlling for age. While a recent study reported associations of 5xSTS time with MVPA in older women,[88] the 5xSTS has been previously reported as an assessment of functional transfer abilities related to a variety of physical and psychosocial factors,[43] explaining why weekly MVPA alone was not strongly associated with this movement quality measure. Another possible explanation for the lack of association between 5xSTS time and physical activity participation is that the nature of the physical activity was unknown; activities like climbing stairs are more likely to carry over to transfer abilities than activities like walking.

### 4.1. Limitations

The present analysis was a cross-sectional study of overweight and obese older adults enrolled in an exercise intervention clinical trial.[38] The cohort was about as active as the general population of older adults, however is likely more active than the general population of overweight and obese adults; about 50% of participants in this analysis did not meet CDC and WHO physical activity recommendations[59, 60] allowing for associations between physical activity and movement quality metrics to be identified.

Hergenroeder et al. showed Actigraph activity monitors can underestimate step count in slower walkers, especially those walking slower than 0.80 m/s.[89] While this finding may impact activity counts as well, the participants in this analysis ambulated with a median gait speed of 1.08 m/s with an IQR of 0.25 m/s. Though it is possible the association between gait speed and physical activity could have been impacted by decreased activity counts among slow walkers, nearly all participants in this analysis walked with gait speeds greater than 0.80 m/s and thus were unlikely to be affected by this phenomenon.

Due to the cross-sectional nature of this analysis, it is unclear if the movement quality metrics are a cause or effect of physical activity participation. Future work should explore the direction of this association. Additionally, this analysis only explored if these associations existed among older adults with high BMIs. Being overweight or having obesity has been shown to affect movement quality in adults of all ages[90–93] and aging has been linked to deficits in gait, balance, and functional mobility in adults across the BMI spectrum.[77, 78, 94] The musculoskeletal, physiological, and psychosocial factors typical in aging adults and adults with obesity were not explored in this analysis. Future work should evaluate these factors to better understand the mechanisms behind the associations observed. Further, middle age has been shown to be a time of disability development.[95, 96] Future work should explore if the relationships observed in this analysis hold for overweight and obese middle-aged adults. Identifying the association between these movement quality metrics and weekly physical activity participation in middle-aged adults can help promote targeted interventions even earlier in the disability development continuum if necessary to promote healthy aging.

## 5. Conclusions

Unstructured physical activity participation is associated with movement quality in overweight and obese older adults. All of the associations observed in this analysis indicate better dynamic balance and functional mobility with greater physical activity. These measures of movement quality have also been previously linked to lower incidence of falls and disability development. Older adults with high BMIs who are less active also demonstrate poorer movement quality which should be targeted in interventions to promote healthy aging.

## Figures and Tables

**Table 1:** Participant demographics and self-reported function and disability. All values presented as median (interquartile range) unless otherwise noted.

Weekly MVPA (min)	158.3 (213.6)
Sex - Female, (n (%))	101 (64.7)
Age (years)	77.0 (8.0)
Height (cm)	165.1 (13.7)
Weight (kg)	83.1 (19.4)
BMI (kg/m^2^)	29.2 (5.3)
26 ≤ BMI < 30 Overweight, n (%)	89 (57.1)
BMI ≥ 30 Obese, n (%)	67 (42.9)
LLFDI - Function (out of 100)	59.9 (10.8)
LLFDI - Disability (out of 100)	77.6 (19.1)

MVPA: moderate-vigorous physical activity, BMI: body-mass index, LLFDI: Late-Life Function & Disability Instrument

**Table 2:** Movement quality measures for all participants presented as median (interquartile range).

Gait speed (m/s)	1.08 (0.25)
Stride length (cm)	118.27 (21.18)
Double-limb support time (% gait cycle)	32.00 (4.88)
Harmonic Ratio - ML ^a^	1.79 (0.68)
Harmonic Ratio - V ^a^	2.18 (0.95)
Harmonic Ratio - AP ^a^	2.74 (1.19)
Figure of 8 Walk Test (s)	9.56 (2.89)
Figure of 8 Walk Test (steps)	17 (4)
Five-Times Sit to Stand (s) ^b^	12.96 (3.86)

a n = 147

b n = 151

ML: medial-lateral, V: vertical, AP: antero-posterior

**Table 3:** One-tailed Spearman’s rank correlation (ρ) of movement quality measures with weekly minutes of MVPA controlling for age.

		MVPA/week (min)		
		ρ_df_	*P*	Post-hoc power
age	**Gait speed (m/s)**	**0.301_153_**	**< 0.001**	0.986*
	**Stride length (cm)**	**0.326_153_**	**< 0.001**	0.995*
	**Double-limb support time (% gait cycle)**	**−0.257_153_**	**< 0.001**	0.947*
	Harmonic Ratio - ML	0.074_144_	0.187	0.226
	**Harmonic Ratio - V**	**0.167_144_**	**0.022**	**0.650**
	**Harmonic Ratio - AP**	**0.140_144_**	**0.046**	**0.521**
	**Figure of 8 Walk Test (s)**	**−0.223_153_**	**0.003**	0.879*
	**Figure of 8 Walk Test (steps)**	**−0.221_153_**	**0.004**	0.874*
	Five-Times Sit to Stand (s)	−0.092_148_	0.132	0.302

**Bolded** indicates *P* < 0.05

* Post-hoc power ≥ 0.80

df: degrees of freedom

ML: medial-lateral, V: vertical, AP: antero-posterior

## Data Availability

Data is available upon request.
